# Cross-TCR Antagonism Revealed by Optogenetically Tuning the Half-Life of the TCR Ligand Binding

**DOI:** 10.3390/ijms22094920

**Published:** 2021-05-06

**Authors:** Omid Sascha Yousefi, Matias Ruggieri, Vincent Idstein, Kai Uwe von Prillwitz, Laurenz A. Herr, Julia Chalupsky, Maja Köhn, Wilfried Weber, Jens Timmer, Wolfgang W. A. Schamel

**Affiliations:** 1 Faculty of Biology, University of Freiburg, 79104 Freiburg, Germany; sascha.yousefi@biologie.uni-freiburg.de (O.S.Y.); matias.ruggieri@biologie.uni-freiburg.de (M.R.); Vincent.Idstein@web.de (V.I.); laurenz.herr@medizin.uni-freiburg.de (L.A.H.); juliach@gmx.net (J.C.); maja.koehn@bioss.uni-freiburg.de (M.K.); wilfried.weber@bioss.uni-freiburg.de (W.W.); 2 Signalling Research Centres BIOSS and CIBSS, 79104 Freiburg, Germany; jeti@fdm.uni-freiburg.de; 3 Center of Chronic Immunodeficiency CCI, University Clinics and Medical Faculty, 79110 Freiburg, Germany; 4 Spemann Graduate School of Biology and Medicine (SGBM), University Freiburg, 79104 Freiburg, Germany; 5 Department of Pathology, Faculty of Medicine, University of Freiburg, 79110 Freiburg, Germany; 6 Institute of Physics, University of Freiburg, 79104 Freiburg, Germany; kai.von.prillwitz@fdm.uni-freiburg.de; 7 Freiburg Center for Data Analysis and Modeling (FDM), University of Freiburg, 79104 Freiburg, Germany

**Keywords:** antagonism, signaling, TCR, T cell activation, modeling, synthetic biology

## Abstract

Activation of T cells by agonistic peptide-MHC can be inhibited by antagonistic ones. However, the exact mechanism remains elusive. We used Jurkat cells expressing two different TCRs and tested whether stimulation of the endogenous TCR by agonistic anti-Vβ8 antibodies can be modulated by ligand-binding to the second, optogenetic TCR. The latter TCR uses phytochrome B tetramers (PhyBt) as ligand, the binding half-life of which can be altered by light. We show that this half-life determined whether the PhyBt acted as a second agonist (long half-life), an antagonist (short half-life) or did not have any influence (very short half-life) on calcium influx. A mathematical model of this cross-antagonism shows that a mechanism based on an inhibitory signal generated by early recruitment of a phosphatase and an activating signal by later recruitment of a kinase explains the data.

## 1. Introduction

T cells are activated through their T cell antigen receptor (TCR), which binds to foreign peptides presented on MHC molecules of the body’s own cells. These peptides are usually derived from bacteria, viruses or other pathogens, but can also be mutated self-peptides in case of tumor cells. Peptide-MHCs to which T cells respond strongly and mount an immune response are called agonists. Binding of the agonist to the TCR stabilizes the TCR in an active conformation enabling intracellular signaling to occur [1,2]. One hallmark of T cell signaling is calcium influx from the extracellular space to the cytosol, which is a TCR proximal signaling event.

Agonists have a high affinity to the TCR. Mutations in the peptide result in ligands of lower affinity that are known as altered peptide ligands [3]. They can be classified into partial agonists, antagonists, and null peptides [4,5]. Partial agonists have intermediate affinities to a given TCR and induce weak or partial T cell responses. Antagonists are of lower affinity and do not elicit any response by themselves, but reduce T cell activation when presented concomitantly with the agonistic peptide [5]. Null peptide-MHCs do not bind to the TCR and do not have an effect on T cell activation.

There is substantial interest in understanding of how the antagonists work, since mutated peptides that inhibit T cell activation have been identified in infections, such as malaria, HIV and hepatitis B and C, and might be involved in evading an immune attack [5].

In general, antagonists could act by three different mechanisms [5]. (i) The antagonist and agonist compete for binding to MHC. In order to inhibit the antagonist has to be loaded on the MHC of the presenting cell in 100–1000-fold excess over the agonist [4,5]. Thus, it might be that the antagonistic peptide displaces the agonistic one from being present on MHC. (ii) The antagonist and agonist compete for binding to the TCR. In excess, the antagonist would block TCRs from being engaged by the agonist [4,6,7]. (iii) The antagonist stimulates the TCR to generate a negative intracellular signal that is capable of inhibiting the activating signal that the agonist induces [8,9,10,11]. These three models are not mutually exclusive and only the last one would allow antagonists to inhibit T cell activation by the agonist at similar concentrations.

To test the model of the inhibitory signal, T cells have been used that express two different TCRs, so that the agonist binds to one and the antagonist to the other TCR. Since the two peptides also bind to different MHC molecules, this set-up eliminates any possibility of competition between agonistic and antagonistic peptides for binding to MHCs or to TCRs. An antagonism in this system is called cross-antagonism. Cross-antagonism is in favor of the inhibitory signal elicited by the antagonist. However, the experiments gave opposing results, in that cross-antagonism was either reported [8,12] or not [6,7], depending on the readout used [13] and the number of available TCRs [14]. The readouts were all very downstream of TCR engagement, such as T cell proliferation or cytokine production, so that additional mechanisms might have confounded the interpretation. Thus, it would be preferable to test cross-antagonism using a proximal TCR signaling readout.

Whether a peptide-MHC acts as an agonist or not depends on the peptide-MHC-TCR affinity [15,16,17,18]. Moreover, different antibodies towards the TCR have been described to either act as agonistics or antagonistics [19]. Since the affinity often correlated with the half-life of the interaction, the kinetic proofreading (KPR) model was proposed [20]. This model suggests that a long half-life of the interaction, such as seen for agonists, allows a series of biochemical reactions to be completed at the TCR that eventually trigger intracellular signaling. By contrast, a low affinity ligand detaches before an activating signal is produced and the TCR then reverts quickly to the initial inactive state, thus not initiating T cell activation. However, mutating the peptide to obtain different affinities and half-lives might also change other biophysical parameters, such as the free binding energy, on-rate [21,22,23], geometry of the interaction [24], conformational changes at the TCR [25,26,27,28] or the ability to withstand pulling forces [29,30]. Thus, it is difficult to rule out that those parameters determine the activity of a ligand.

Recently, we engineered an optogenetic system in which the half-life of the ligand-TCR interaction could be changed without altering the other parameters [31]. We made use of the plant photoreceptor phytochrome B (PhyB) that binds to the PhyB-interacting factor (PIF) when illuminated with 660 nm light [32,33,34,35]. In our system, PhyB served as a ligand for the GFP-PIF^S^-TCR, in which GFP and the PhyB-binding portion of PIF were fused to the TCR. In addition, we mutated the PIF part, to allow efficient transport through the secretory pathway, and named it subsequently PIF^S^. Importantly, the intensity of 660 nm light determines the cycling rate of PhyB between the PIF binding and non-binding states [36,37] and, thus, the half-life of the ligand-TCR interaction. We showed that the half-life alone can determine whether a TCR ligand triggers intracellular signaling or not [31], in that a half-life of 8 s or longer was needed to induce optimal signaling, as measured by calcium influx. Similar results were obtained using a different optogenetic system [38], corroborating our conclusions. These elegant approaches may also be highly suitable to study antagonism yet.

Here, we tested the ability of the optogenetic TCR to cross-antagonize a second unrelated TCR as a function of the half-life of the opto-ligand-TCR interaction. We hypothesized that a short half-life would diminish the agonist signal of the second TCR, whereas a long half-life would not, and this was indeed the case. A mathematical model also showed that cross-antagonism based on the half-life is a possible mechanism to downregulate T cell responses. Finally, our study shows again that engineering TCRs delivers new insight into TCR function as it can also be used for cancer immunotherapy [39,40].

## 2. Results

### 2.1. Characterization of Dual TCR Expressing Cells

**Figure 1:**
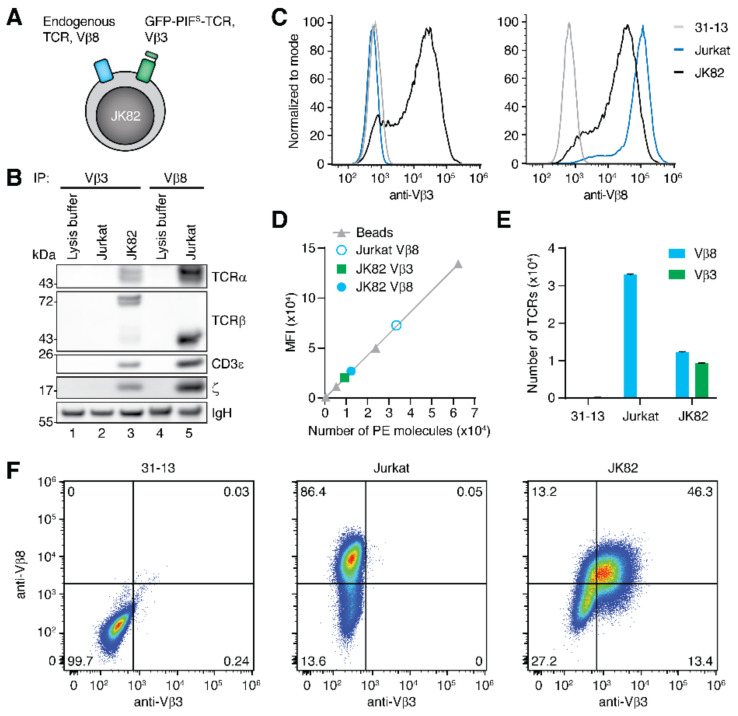
Analysis of the number of Vβ3 and Vβ8 TCRs on the cell surface. (**A**) Scheme of the JK82 cells expressing the endogenous Vβ8 TCR and the transduced GFP-PIF^S^-TCR containing a Vβ3 TCRβ chain. (**B**) Lysis buffer, Jurkat or JK82 cell lysates were used to immuno-precipitate TCRs with either anti-Vβ3 or anti-Vβ8 antibodies. Purified proteins were separated by SDS–PAGE and the Western blot developed for the TCR subunits TCRα, TCRβ, CD3ε, and ζ. (**C**) JK82, Jurkat, and 31–13 cells were stained with saturating amounts of anti-Vβ3 or Vβ8 antibodies labeled with PE and analyzed by flow cytometry. (**D**) Quantibrite beads coupled to defined amounts of PE molecules were measured in a flow cytometer to generate a linear relation of numbers of PE molecules versus PE fluorescence (given as mean fluorescence intensity, MFI). The values of the MFI measurement in (**C**) only considering the PE-positive cells as well as the calculated number of PE molecules is shown as well. (**E**) The graph shows the calculated total number of TCRs on the cell surface per cell of 31–13, Jurkat, and JK82 cell lines. Error bars represent the SEM (*n* = 4−5). (**F**) 31–13, Jurkat and JK82 cells were co-stained with anti-Vβ3 (eFluor405) and anti-Vβ8 (PE) antibodies and analyzed by flow cytometry. One representative experiment of *n* = 3 is shown.

In order to study cross-antagonism, we needed cells that express two different TCRs. One TCR should contain the GFP-PIF^S^-TCRβ chain, so that our optogenetic approach could be used to vary the half-life of the receptor-ligand interaction [31]. The GFP-PIF^S^-TCRβ chain that contains a Vβ3 domain was lentivirally expressed in Jurkat cells that contained their endogenous Vβ8 TCR (Figure 1A). For simplicity these dual expressing cells are named JK82 (Figure 1B). To show that the GFP-PIF^S^-TCRβ chain assembled with the TCRα and CD3 subunits in these cells, JK82, and as a control, wild type Jurkat cells were lysed, and an anti-Vβ3 immuno-purification was performed (Figure 1B). Reducing SDS–PAGE and Western Blotting show that the GFP-PIF^S^-TCRβ chain has the expected size of approximately 72 kDa and that TCRα, CD3ε (representing the CD3 heterodimers), and ζ were co-purified (lane 3). Because ζ is the last subunit to be added to the TCR complex during assembly [41,42], its presence indicates that a complete TCR has formed. The control anti-Vβ3 purification from Jurkat lysates did not reveal any TCR subunit (lane 2). We also performed an anti-Vβ8 immuno-purification from Jurkat cells and showed that the endogenous Vβ8 chain has the expected size of 40 kDa and co-purified TCRα, CD3ε, and ζ as well (lane 5). GFP-PIF^S^-TCRβ was expressed on the Jurkat cell surface (Figure 1C), again showing that it assembled to a complete TCR.

Next, we quantified the amounts of the GFP-PIF^S^-TCR and the Vβ8 TCR on the surface of the JK82 cells. JK82, Jurkat and 31–13 cells were stained with saturating concentrations of PE-labelled anti-Vβ3 (Figure 1C left panel) or anti-Vβ8 antibodies (right panel). The 31–13 cells are Jurkat derived and lack the expression of a TCRβ chain, and thus do not contain any TCR on the cell surface. Flow cytometry showed that only JK82 cells displayed a Vβ3 TCR on their surface (left panel) and that JK82 and Jurkat cells displayed a Vβ8 TCR (right panel). In parallel, beads with a defined number of PE molecules were measured as well and their mean fluorescence intensity (MFI) is in linear relation to the number of PE molecules that they contain (Figure 1D). Thus, from the MFI of the JK82 and Jurkat cells, we can estimate the number of TCRs. Jurkat cells contained about 33,000 Vβ8 TCRs, and JK82 expressed approximately 12,000 Vβ8 TCRs and 9100 Vβ3, i.e., GFP-PIF^S^-TCRs (Figure 1E). Hence, the total number of TCRs was lower in the JK82 compared to the parental Jurkat cells, which might be due to less effective expression/folding/assembly of GFP-PIF^S^-TCRβ compared to the endogenous Vβ8 chain. Nevertheless, both TCRs are expressed to similar levels in JK82 cells (Figure 1E) and on the same cell as seen in the dual staining (Figure 1F).

### 2.2. Stimulation of JK82 Cells with Agonistic Anti-Vβ8 Antibodies and PhyBt

**Figure 2:**
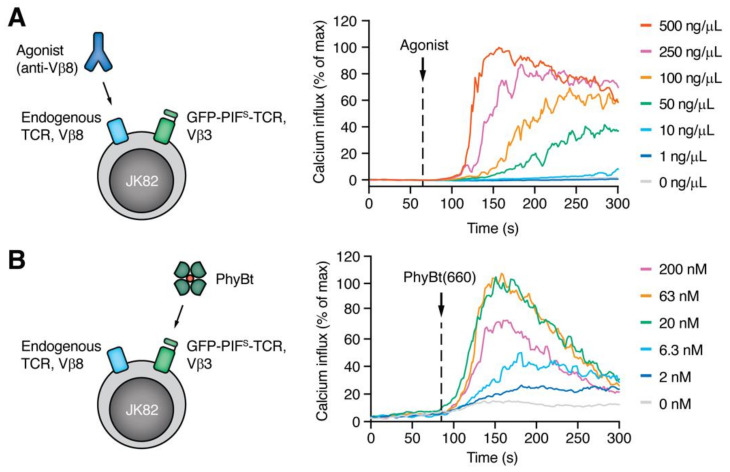
TCR ligand titration. Calcium influx into JK82 cells was measured upon treatment with different concentrations of anti-Vβ8 (**A**) or with PhyBt (**B**). PhyBt was pre-illuminated with 660 nm light (PhyBt(660)) to switch PhyB to the GFP-PIF^S^-TCR-binding conformation. One representative experiment of *n* = 3 is shown.

Our aim was to stimulate the Vβ8 TCR with anti-Vβ8 antibodies as the high affinity agonist ligand and optogenetically manipulate the half-life of the interaction of GFP–PIF^S^-TCR with PhyB tetramers (PhyBt). The anti-Vβ8 antibody was used as an artificial TCR ligand and a model agonist and will from now on be referred to as “agonist”. In order to see additive or antagonistic effects on the agonistic signal by stimulating the GFP–PIF^S^-TCR, we needed to use an agonist concentration that induced intermediate calcium influx levels. By titrating the agonist between 500 and 1 ng/µL we found that 50 ng/µL led to intermediary calcium influx (Figure 2A), which we will use in all upcoming experiments.

Additionally, we titrated PhyBt to find optimal stimulation conditions. To this end, we purified light-responsive biotinylated PhyB monomers that were produced in *E. coli* as described [43]. Streptavidin-based PhyB tetramers were formed and purified as before [44]. PhyBt was pre-illuminated with saturating amounts of 660 nm light, called PhyBt(660), resulting in a steady state, in which 80% of the PhyB molecules are in the conformation, in which they can bind to the GFP-PIF^S^-TCR [31]. Moreover, 63 and 20 nM of PhyBt(660) showed the highest calcium influx (Figure 2B) and will be used henceforth, if not stated otherwise. With 200 nM PhyBt(660) the calcium response was reduced compared to 63 nM, due to decreased multivalent TCR binding at the very high ligand concentrations [31].

### 2.3. Modulation of Agonistic Stimulation by Different Ligand Binding Half-Lives to the GFP-PIF^S^-TCR

To experimentally control the half-life of the ligand-TCR interaction, we exploited the property of PhyB that a continuous exposure to 660 nm light triggers both: the switch from the non-PIF binding to the PIF-binding state and the reverse one from the binding to the non-binding state [31,36,37]. Thus, each individual PhyB molecule constantly shuttles between these two conformational states under 660 nm light, with the light intensity determining the shuttling rate. The intensity does not alter the number of PhyB molecules that are in the binding state at any moment; in fact at any 660 nm light intensity and at any time point 80% of the PhyBs are in the binding state [34,36]. Thus, increasing the 660 nm intensity decreases the half-life of the PhyB–GFP-PIF^S^-TCR interaction, but not the number of PhyB molecules binding to the GFP-PIF^S^-TCR. Of note, the 80% do not bind constantly, but are part of all PhyBs that shuttle between the binding and non-binding states.

**Figure 3:**
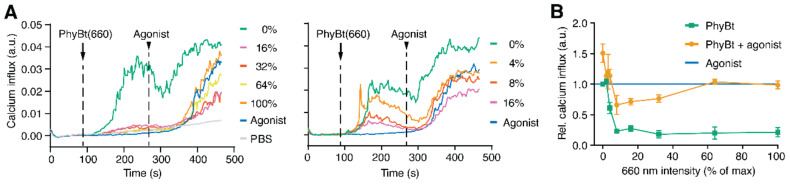
Cross-modulation of agonist stimulation using optogenetically controlled ligand-binding times. (**A**) JK82 cells were first treated with 63 nM PhyBt(660) (arrow) under different 660 nm light intensities as depicted and then in addition with 50 ng/mL of the agonist anti-Vβ8 (arrowhead) while measuring calcium influx using flow cytometry. Additionally, cells were treated with PBS only (arrow and arrowhead) or first with PBS (arrow) and then with the agonist at the arrowhead time point. One representative experiment of *n* = 13 is shown. (**B**) The relative calcium influx was quantified from experiments as in (**A**) for the PhyBt(660) treatment alone (green rectangles) or combined with the agonist (orange dots) from 13 different experiments as described in the methods. The blue horizontal line indicates stimulation by the agonist alone.

Having this in mind, we studied the effect of varying ligand-binding half-lives of our optogenetic GFP-PIF^S^-TCR on agonistic stimulation by altering the intensities of 660 nm light. We measured calcium influx into the cells using flow cytometry to monitor T cell stimulation (Figure 3A). We first added PhyBt(660) under continuous 660 nm light and quantified calcium influx for three minutes (arrow). This stimulation led to decreasing calcium influx with increasing 660 nm light intensity (Figure 3A,B, green line in B), that is with decreasing ligand binding half-lives. This result is in line with our previous finding that T cells employ a KPR mechanism, thereby responding to long ligand binding whilst not being activated by short ligand binding [31]. While still quantifying calcium influx, we added the agonist (anti-Vβ8, arrowhead) and then measured for another three minutes (Figure 3A). Light intensities below 8% showed an additive effect on the calcium influx when compared to the agonist alone (Figure 3A,B, orange line in B; 8% refers to the maximum intensity (100%) that our light device can generate). This suggests that long ligand binding half-lives enhance or add towards agonist-induced signaling. In contrast, intensities between 8% and 32% resulted in reduced calcium influx compared to agonist stimulation alone, indicating that signaling by short half-life-stimulated TCRs can inhibit signaling by the agonist stimulated TCRs. Above 32%, no change compared to agonist stimulation alone was seen (Figure 3A,B, orange line in B). Consequently, very short ligand binding times displayed neither enhancing nor inhibiting effect on agonist stimulation.

In conclusion, this experiment shows that cross-antagonism is dependent on the half-life of the ligand-TCR interaction. The same conclusion was reached when we added PhyBt(660) and the agonistic anti-Vβ8 simultaneously (Figure S1), and not sequentially as above.

### 2.4. Mathematical Model of Cross-Antagonism

Having shown that the half-life of ligand-TCR interaction determines whether a ligand is an antagonist or not, we aimed to obtain further insight by a mathematical model. The model is based on kinetic proofreading (KPR) models [20,45,46,47] and assumes activation of the phosphatase SHP1 by the inhibitory state and activation of the tyrosine kinase ZAP70 by the activatory state. Indeed, SHP1 has experimentally been shown to be involved in antagonism [10,11] and ZAP70 is the main activatory kinase recruited to the TCR [45,46].

**Figure 4:**
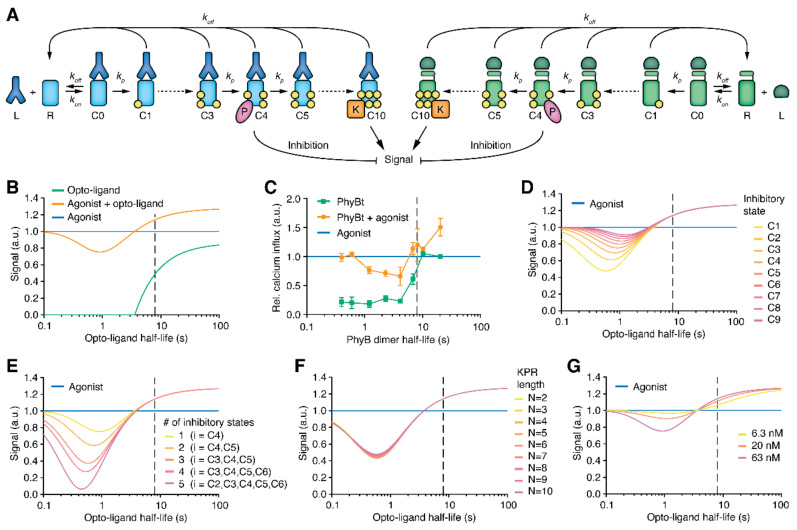
Mathematical model. (**A**) Schematic representation of the C10 Signal Inhibition model, combining two KPR schemes, one for each ligand-TCR pair (left: agonist; right: opto-ligand). The activating signals by the final state C10 can be reduced by inhibitory signals induced by the intermediate state C4. Ligand-receptor association rate $k_{on}$ and KPR step rate $k_{p}$ are the same for both submodels. The dissociation rate $k_{off}$ is submodel-specific. (**B**) The signal vs opto-ligand binding half-life for each ligand alone and for their combination was calculated by the C10 Signal Inhibition model. (**C**) Data from Figure 3B with the 660 nm intensity converted to ligand binding half-life. (**D**–**G**) C10 Signal Inhibition model results varying the position of the inhibitory state (**D**), the number of inhibitory states (**E**), the number of KPR steps (**F**), or the opto-ligand concentration (**G**). In (**F**), the state C1 has been used as the inhibitory state. The opto-ligand concentration in (**B**–**F**) is 63 nM. The dashed line in (**B**–**G**) marks $\tau_{KPR}=8\;s$.

In the KPR model, after ligand binding, a series of *N* (here N=10) modification steps must be completed before the TCR can trigger an activatory signal in the last state, i.e., in state C10. In each state, the ligand can dissociate, leading to instant reversal of all so far attained modifications (Figure 4A). Hence, a ligand’s ability to trigger activation is directly related to the half-life of the ligand-TCR complex [20]. As before [48], we implemented an intermediate state that elicits an inhibitory signal (Figure 4A, state C4), that acts on the activatory signal. In our model, the inhibitory signal is subtracted from the activating signal to give the final signal (that we measured as calcium influx). We refer to this version of the model as the C10 Signal Inhibition model (details on the model are found in the supplement; Figure S2 and Supplemental Information). Since the two ligands do not compete for the same receptor, we have two independent KPR models for the two ligand-TCR pairs that are only combined at the signal output level.

Biologically, the activating signal involves kinases, such as Lck or ZAP70, and the inhibitory signal the phosphatase SHP1 (note that the mathematical model itself is independent of this interpretation). Since phosphatases are more active than kinases, we introduced a factor *f* (f=20) that amplifies the inhibitory signal relative to the activatory signal before subtraction. In the model, due to the long binding time of the agonist-TCR complex (small off-rate in Table S2), almost all agonist TCRs are in the final signaling state and the inhibitory effect of the agonist itself is thus negligible.

A crucial parameter of KPR is the threshold half-life, $\tau_{KPR}$, of the ligand-TCR complex that is required to produce a significant signal. In the basic KPR model without inhibition, $\tau_{KPR}$ is defined as the half-life for which half of the bound receptors are in state C10. The model was parametrized with $\tau_{KPR}=8\;s$ [31].

The C10 Signal Inhibition model was able to produce the expected behavior of the final signal (calcium influx) as function of the half-life of the opto-ligand-TCR interaction (Figure 4B). The signal that the agonist alone elicits is set to 1.

Firstly, simulating the activity of the opto-ligand alone (Figure 4B, green line) showed that the ligand was only activatory above a certain binding half-life, as we reported before [31]. Secondly, in the cross-antagonism simulation, the opto-ligand acted as a co-agonist for long half-lives, enhancing the signal produced by the agonist alone (Figure 4B, orange line). For intermediate half-lives, the opto-ligand acted as antagonist and, while producing no signal by itself, reduced the agonist signal. For even smaller half-lives, the opto-ligand was non-stimulatory. These findings are in good agreement with the experiment (Figure 4C). One discrepancy was, however, that the opto-ligand-TCR half-life, for which the antagonistic effect was strongest, was smaller in the model than in the experiment (for a possible explanation see below).

### 2.5. Predictions of the Model

Next, we varied the position and amount of inhibitory states in the C10 Signal Inhibition model and simulated the output signal (Figure 4D,E). We found that the antagonistic effect of the opto-ligand increased when the single inhibitory state was moved to an earlier position in the KPR chain, or when the number of inhibitory states was increased. However, the half-life for which the strongest inhibition was observed moved to even lower values, increasing the discrepancy between model and experiment.

Recent results suggest that the number of relevant KPR modifications might be as small as N=2 or 3 [38,49]. We thus varied the number of KPR steps in our model and found that the model’s result is extremely robust upon this variation (Figure 4F). This can at least partially be explained by an adjustment of the KPR step rate $k_{p}$ that had to be done in order to keep $\tau_{KPR}$ fixed to 8 s; and the induced adjustment of $k_{p}$ almost cancel each other out, so that the difference between the amount of receptors in the final signaling state and in the inhibitory state (amplified with the inhibition factor f) is almost unaffected. We used the first receptor state C1 as the inhibitory state (instead of C4), which allowed us to reduce the KPR length down to N=2. We verified that the robustness of the model result upon variation of N also holds for other positions of the inhibitory state (not shown).

The C10 Signal Inhibition model could also be used to make predictions for variations of parameters that can be varied experimentally. In the basic KPR model the threshold half-life, $\tau_{KPR}$, is largely independent of the ligand concentration [20], and this was also the case in our optogenetic ligand-TCR system [31]. Thus, we simulated the dependence of the antagonistic effect on the concentration of the opto-ligand. We found that the antagonistic effect at intermediate binding half-lives was reduced for an opto-ligand concentration of 20 nM compared to 63 nM, and almost vanished for a concentration of 6.3 nM (Figure 4G). This will be experimentally validated below.

Finally, we tested an alternative model with a different mechanistic implementation of the inhibition. Following previous work [47,50], the inhibitory state activates the phosphatase which induces a backwards reaction rate of all the KPR steps (Figure S3). The model output consisted now only of the activatory signal triggered by the final signaling states C10. For the ability of the opto-ligand to inhibit the agonist, it is crucial that both ligand-TCR pairs interact with the same phosphatase. A full description of this model (the KPR Steps Inhibition model) is given in the Supplement. The underlying biological assumption is that the different receptor types are mixed on the cell surface, either because the receptors are localized within the same nanocluster [51,52,53] or because they rapidly diffuse and meet each other. Simulation of this model revealed that it could also produce the expected qualitative result, namely that the opto-ligand can act as a co-agonist, antagonist or null-ligand depending on its binding half-life (Figure S4). For the used parametrization, the comparison to the C10 Signal Inhibition model showed that the maximal antagonistic effect of the opto-ligand was weaker in the KPR Steps Inhibition model. However, the opto-ligand-TCR half-life for which the maximal antagonistic effect was observed was now larger, reducing the discrepancy between model and experiment.

### 2.6. Cross-Antagonism Depends on the Concentration of the Antagonistic Ligand

**Figure 5:**
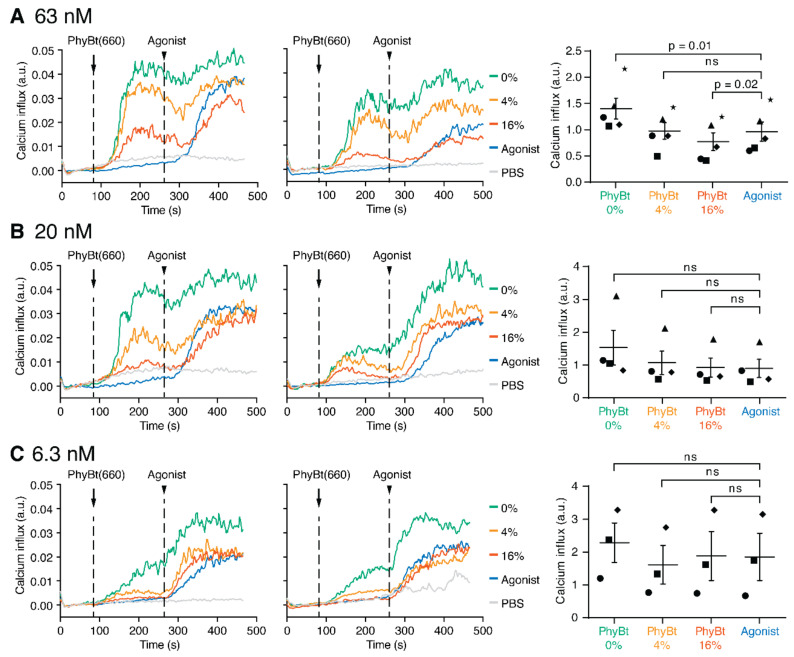
The antagonist concentration determines the extent of cross-antagonism. (**A**) JK82 cells were first stimulated with 63 nM PhyBt(660) (arrow) under 0%, 4% or 16% 660 nm light intensities and then with 50 ng/mL agonist (anti-Vβ8, arrowhead) while measuring calcium influx using flow cytometry. Additionally, cells were treated with PBS or agonist alone as in Figure 3. (**B**) As in (**A**), but using 20 nM PhyBt. (**C**) As in (**A**), but using 6.3 nM PhyBt. Two representative experiments of *n* = 5 (in **A**), *n* = 4 (in **B**) and *n* = 3 (in **C**) as well as the quantification of all experiments are shown. The different symbols of the quantification depict different experiments.

Our mathematical model predicted a dependence of the cross-antagonistic effect on the PhyBt(660) concentration (Figure 4G). To validate this prediction, we repeated the sequential PhyBt(660) and agonist stimulation experiments as in Figure 3 using 0%, 4%, and 16% 660 nm light intensities and either 63, 20, or 6.3 nM PhyBt(660) concentrations (Figure 5A–C, respectively). Using 63 nM PhyBt(660), all performed experiments showed a lower calcium influx under 16% light intensity compared to agonist treatment alone (Figure 5A), again demonstrating that cross-antagonism exists in our experimental system. However, with 20 nM and 6.3 nM PhyBt(660), the antagonistic effect at 16% 660 nm light intensity was not seen (Figure 5B,C). These results clearly validate the prediction of our mathematical model.

## 3. Discussion

Here, we demonstrate that the half-life of the ligand-TCR interaction determines whether a ligand acts as a co-agonist, antagonist, or does not have any effect on TCR signaling as quantified by calcium influx. Without experimental evidence mathematical models have already proposed that the half-life determines the agonist and antagonist properties of the TCR ligand [47,48,50,54,55]. Indeed, correlations of the half-life and the antagonistic nature of a TCR ligand have been reported [17,18,56,57]. With our optogenetic approach it was possible to change the binding half-life of the ligand without changing other binding parameters such as the on-rate, free binding energy, geometry of the interaction, conformational changes at the TCR or the ability to withstand pulling forces [31]. This is not possible by mutating peptide-MHC, i.e., by mutating the ligand-TCR interface, since this strategy changes several parameters at once. In our optogenetic system, the half-life of the ligand was only changed by the intensity of illumination using the same unaltered protein-protein pair for each half-life variation. Thus, other possible parameters of the ligand-receptor interaction were unaffected, proving that the half-life of the ligand-TCR interaction is the critical factor.

We used the opto-ligand-TCR system before to show that T cells distinguish between activatory (agonistic) and non-activatory ligands based on the ligand binding half-life to the TCR [31] as has been proposed by McKeithan’s KPR model.

Due to two different TCRs and two different ligands, we exclude competition of the agonist and antagonist in binding to the TCR, thus proving that cross-antagonism can occur, as has been reported before using peptide-MHC [8,12,13,14]. This implies that the antagonistic effect is based on the generation of an inhibitory signaling event by the receptor that binds to the antagonist that counteracts the activating signal evoked by the receptor that is agonist-bound. Since the phosphatase SHP1 plays a role in antagonism [8,10,11], it is likely that SHP1 is a key enzyme in this inhibitory signal by counteracting phosphorylation events by the kinases Lck and ZAP70 that constitute the activating signal [1,45,46].

Our agonist was an anti-Vβ8 antibody, thus being of very high affinity compared to typical high affinity peptide-MHC. Despite this, we could antagonize the signal it provoked. This is in line with previous studies using antibodies or peptide-MHC as the antagonist [19,58]. Further, it was proposed that the immunological synapse was involved in generating the antagonistic signal by assembling into a non-activating configuration [56]. However, in contrast to peptides loaded on MHC on antigen presenting (or target) cells, soluble ligands—as we have used here—do not lead to the formation of a synapse. Hence, a synapse is not necessary for the generation of the antagonistic signal. Likewise, the agonistic activating signal does not need a synapse to form [59,60]. In fact, agonistic TCR signaling is upstream of and hence required for synapse formation, suggesting that antagonists inhibit the signal that is necessary for the formation of an activating synapse.

Considering the mechanistic insight detailed in the previous paragraphs, we developed a mathematical model on cross-antagonism based on KPR models [20,48,55]. Our C10 Signal Inhibition model is different from previous antagonism models [50,55,61] and captures our data well. Our experiments show that the ligand binding half-life ($\tau_{KPR}$ = 8 s) determined whether one ligand given alone is stimulatory or not. This half-life coincided with the half-life that determined whether a second ligand, which was applied together with a strong agonist, acted as an antagonist or not. Above this threshold, the ligand served as a co-agonist and below the antagonist feature manifests. This suggests that a similar molecular mechanism is involved. In the C10 Signal Inhibition model, this observation is the consequence of the kinetic independence of the two ligand-TCR pairs and the calculation of the net signal as a difference of the positive and negative signal. At the threshold between (co-)agonistic and antagonistic behavior, the positive and negative signal exactly cancel each other out. In this case, the second ligand neither had an effect when applied alone nor when applied together with the agonist. When the second ligand alone produced a positive net signal, this signal would enhance the agonist signal in the case where both ligands were applied. When the second ligand produced a negative net signal, this signal would inhibit the agonist signal.

The C10 Signal Inhibition model allowed us to simulate changes in the antagonistic proofreading schemes that could not be addressed experimentally. Firstly, we changed the position of the inhibiting state within the 10 states that constitute our KPR chains. The earlier the inhibitory state was placed, the more pronounced the inhibitory effect was. This was explained by the fact that ligands constantly dissociate from the TCRs and the earlier the inhibitory state occurred the more often it was visited, i.e., the more receptors were in this state at any given moment.

Secondly, the higher the number of intermediate states that were inhibitory, the stronger the inhibitory effects were. Again, the larger the number of TCRs that were in an inhibitory state, the bigger the antagonistic effect was. Hence, in our model, the relative abundance of the inhibiting and activating states determines the total output.

Finally, we were able to improve our model by not only allowing the inhibitory state to act on the signal emitted by the final activating state C10, but by acting on all transitions from one state to the other (KPR Steps Inhibition model). A similar arrangement was also used in a previous model [47]. In the C10 Signal Inhibition model, the maximum of the antagonistic effect was seen at very short half-lives (1 s or below), whereas in the KPR Steps Inhibition model the maximal inhibitory effect was at approximately 2–3 s, being more in agreement with the experimental data. Moreover, from the biological point of view, we found the KPR Steps Inhibition model more realistic. Indeed, SHP1 being recruited to a TCR should be able to remove phosphate groups from any state (intermediate or final) and not only from the final one, as we assumed in the C10 Signal Inhibition model.

One important question is how SHP1 is employed by TCRs that are antagonist bound. One mechanism might be that TCRs in the inhibitory state activate SHP1 that then diffuses to the cytosol and acts on the signal emitted by C10 (C10 Signal Inhibition model). Alternatively, SHP1 might be bound to the inhibitory state and remain there, only acting on substrates close to the TCR to which SHP1 is bound, such as the other states of the same TCR (in our case the GFP-PIF^S^-TCR, KPR Steps Inhibition model) or of the other TCR (in our case the agonist-bound Vβ8 TCR). Indeed, TCRs are nanoclustered on the cell surface [51,53] and mixed nanoclusters might form, so that SHP1 at one receptor might dephosphorylate others. Several reports suggest that SHP1 might localize close to the TCR; SHP1 binds to Lck [10] and Lck might come into the vicinity of the TCR by being bound to the co-receptors CD8 and CD4 [62,63], because they themselves bind to the same peptide-MHC as the TCR [64]. Hence, a complex with TCR–peptide-MHC–CD8/4–Lck–SHP1 might form. Thus, it could be that the co-receptors are involved in the generation of the inhibitory signal by bringing SHP1 via Lck to the antagonist-bound TCR, as already implemented into a mathematical model of antagonism [54]. However, a co-receptor was not necessary to produce the antagonistic signal, except if the antagonist was of very low affinity [58]. Our system did not involve any co-receptor, again showing that the negative signal could be generated without CD8 or CD4. We have recently shown that Lck can directly bind to the TCR [65] and thereby might recruit SHP1 to the antagonist-bound TCR without co-receptor involvement. Our data here would support this possibility. Another possibility is that SHP1 might dissociate from the antagonist-bound TCR, but still remain attached to the membrane, hence only acting on membrane-bound substrates such as agonist-bound TCRs, as has been suggested for ZAP70 [66]. Finally, SHP1-bound TCRs might diffuse fast enough within the membrane to encounter and inhibit other TCRs.

In our model also the agonist is able to recruit and activate SHP1 (Figure 4A). This effect is, however, insignificant since most agonist receptors are in the final, activatory signaling state. In line with this, an increase of SHP1 phosphatase activity was seen in the antagonism experiment, but not with the agonist alone [11].

In conclusion, an early recruitment of SHP1 to ligand-bound TCRs and dissociation at later time points when ZAP70 is recruited might be an important mechanism in early T cell signaling. T cells might use this mechanism to measure the quality, i.e., the binding half-life of a ligand, to determine whether to become activated or not. Further, antagonist ligands might make use of this mechanism to dampen responses by agonistic ligands, as it is seen in viral infections [5]. Thus, the dynamic changes in the assembly of the TCR signalosome might be a crucial mechanism with which T cells function and take decisions in the immune system.

## 4. Materials and Methods

### 4.1. Molecular Cloning

The plasmid pOSY082 generated in this study was created using standard molecular cloning techniques, such as polymerase chain reaction, restriction enzyme digestion, and Gibson assembly (Gibson et al., 2009) of 5 fragments: (1) SalI–ClaI fragment of p526A (pCDH-EF1-MCS-T2A-copGFP vector from System Biosciences LLC, Palo Alto, CA, USA); (2) PCR with primers O204 and O205 from p526A; (3) PCR with primers O206 and O197 from pOSY076; (4) PCR with primers O198 and O199 from pOSY076 [31]; and (5) PCR with primers O200 and O207 from pOSY076. The primer sequences are listed in Table S1. The integrity of the plasmid was verified by restriction enzyme digestion and Sanger sequencing (Eurofins Genomics, Konstanz, Germany).

### 4.2. Protein Production and Purification

The production of PhyB_1-651_-AviTag-His_6_ was performed as described before [43]. Briefly, PhyB_1-651_-AviTag-His_6_ was expressed in *E. coli* BL21(DE3) cells and purified from bacterial lysates by affinity chromatography on a Ni-NTA Superflow cartridge (Qiagen, Hilden, Germany) using an Äkta Explorer chromatography system (GE Healthcare, Freiburg, Germany). Subsequently, PhyB tetramers (PhyBt) were formed and purified as described previously [44]. Briefly, Ni-NTA column-purified PhyB_1-651_-AviTag-His_6_ was mixed in a 10:1 molar ratio with DyLight650-conjugated streptavidin (Thermo Fisher Scientific, Darmstadt, Germany) and the mixture was incubated for 2 h at room temperature in the dark. Then, PhyBt were separated from the excess of PhyB monomers by size-exclusion chromatography on a HiLoad Superdex 200 pg column (GE Healthcare, Freiburg, Germany) using PBS with 0.5 mM TCEP as mobile phase. The eluate fractions containing the purified tetramers were pooled and concentrated using Spin-X UF 10k (Corning, Kaiserslautern, Germany) centrifuge concentrators to a final concentration between 0.5 and 2 µM.

### 4.3. Cell Line Generation and Cultivation

Jurkat E6.1 and derived cell lines were cultured in RPMI 1640 medium supplemented with 10% fetal bovine serum (FBS), 2 mM L-glutamine, 10 mM HEPES, 100 U/mL penicillin and 100 µg/mL streptomycin (all Thermo Fisher Scientific, Darmstadt, Germany) at 37 °C in a humidified atmosphere of 5% CO_2_. HEK 293T cells were cultivated in DMEM (Thermo Fisher Scientific, Darmstadt, Germany) supplemented as the RPMI medium at 37 °C in a humidified atmosphere of 7.5% CO_2_.

To generate Jurkat and 31–13 cells stably expressing the GFP-PIF^S^-TCRβ chain (JK82 and 31–13 GFP-PIF^S^-TCR), we used lentiviral transduction as described earlier [31]. Briefly, HEK 293T cells were transfected with the envelope plasmid pMD2 vsvG, the lentiviral packaging plasmid pCMV dR8.74 (both kind gifts from Didier Trono), and the transfer plasmids pOSY082 or pOSY076 by calcium phosphate precipitation. Moreover, 6 h post-transfection the medium was replaced by fresh medium, and the transfected HEK 293T cells were incubated for 48 h. Lentiviral particle-containing HEK 293T supernatant was harvested, filtered through a 0.45 µm filter, and concentrated by centrifugation at 10,000× *g* at 4 °C for 4 h through a 20% (*w/v* in PBS) sucrose cushion supplemented with 1 mM EDTA. After centrifugation, the supernatant was discarded; the viral particles were resuspended in medium using 1/100th of the cell supernatant volume. Jurkat and 31–13 cells were transduced with different dilutions of concentrated lentiviral particles and 48 h after transduction, surface expression and cell viability were analyzed by flow cytometry.

The identity of the Jurkat cells was confirmed by the binding to the antibody C305, which only binds to the endogenous Jurkat TCR [67]. The identity of the HEK 293T cells was not confirmed. All cell lines were routinely tested for mycoplasma and devoid of contamination.

### 4.4. Calcium Influx Measurement

Staining of Jurkat cells with the Ca^2+^-sensitive fluorophore Indo-1 was performed as described before [44]. In short, five million cells were centrifuged for 5 min at 300 *g* and the supernatant was discarded. The cells were resuspended in 1 mL stimulation medium (RPMI 1640 medium supplemented with 1% FBS, 2 mM L-glutamine, 10 mM HEPES, 100 U/mL penicillin and 100 µg/mL streptomycin) with 0.1% (*v/v*) pluronic F-127 and 4 µM Indo-1 AM (all Thermo Fisher Scientific, Darmstadt, Germany) and incubated in the dark for 30 min at 37 °C. The Indo-1 stained Jurkat cells were washed once with stimulation medium, resuspended in 500 µL stimulation medium and kept on ice in the dark until the measurement. For each calcium influx measurement, the cells were diluted 1:20 with stimulation medium pre-warmed to 37 °C and maintained at 37 °C during the event collection on a MACSQuant X flow cytometer. After baseline fluorescence acquisition, purified PhyB tetramers, or anti-Vβ8 (Becton Dickinson, Heidelberg, Germany) were added as described.

For the graphs showing the calcium traces (“Calcium influx (a.u.)” on the y-axis, Figure 3 and Figure 5), the ratio of Ca^2+^-bound to Ca^2+^-free Indo-1 fluorescence minus the average baseline value (20–60 s) was quantified using FlowJo 9 (FlowJo LLC, Ashland, OR, USA). For the graphs depicting the calcium influx as % of max (Figure 2), each curve was normalized to the peak value of the respective experiment. To calculate the relative calcium influx values (a.u.) for the PhyBt alone, average calcium influx values after PhyBt addition (180–240 s) were normalized for each experiment to the PhyBt 0% light intensity sample. To calculate the relative calcium influx values (a.u.) for “PhyBt + agonist”, average calcium influx values after anti-Vβ8 addition (380–440 s) were normalized for each experiment to the agonist alone sample.

### 4.5. Cell Lysis and Immunoprecipitation

Jurkat cells and Jurkat cells expressing the GFP-PIF^S^-TCRβ chain (JK82) were counted and 35 million cells were harvested for each immunoprecipitation by centrifugation for 5 min at 4 °C at 300× *g*. Cell pellets were washed once with ice-cold PBS and, subsequently, lysed on ice for 30 min in lysis buffer (20 mM Tris, 137 mM NaCl, 5% glycerol, 1 mM EDTA, 0.5 mM Na_3_VO_4_, 1 mM PMSF, 10 mM NaF, 1× protease inhibitor cocktail, 0.5% (*v/v*) Brij 96V, 0.5% (*w/v*) digitonin at pH 7.4). Lysates were cleared from debris by centrifugation for 10 min at 4 °C at 16,000× *g*. For each immunoprecipitation, 10 µL 1:1 mixed Protein A and G Sepharose beads (GE Healthcare, Freiburg, Germany) were washed once with PBS, loaded with 5 µg anti-Vβ3 (Ancell, Bayport, MN, USA) or anti-Vβ8 (Becton Dickinson, Heidelberg, Germany) by incubating for 1 h at 4 °C on a rotating wheel and excess antibodies removed by another PBS wash step. Cell lysates were added to the beads and incubated for 3 h at 4 °C on a rotating wheel. Next, beads were pelleted by centrifugation for 2 min at 4 °C at 1000× *g*, the supernatant was discarded, and the beads were washed four times with washing buffer (as lysis buffer, but without digitonin and protease inhibitor cocktail). After the final wash step, beads were pelleted, resuspended in 20 μL sample buffer (106 mM Tris-HCl, 141 mM Tris base, 2% LDS, 10% Glycerol, 0.51 mM EDTA, 0.22 mM Coomassie Blue G250, 0.175 mM Phenol Red, 50 mM DTT, pH 8.5) and incubated for 10 min at 70 °C.

### 4.6. SDS-PAGE and Immunoblotting

The immunoprecipitation eluates were separated by SDS–PAGE and detected by immunoblotting using standard protocols. Briefly, samples were subjected to SDS–PAGE separation, transferred to PVDF membranes for immunoblotting with anti-TCRα (H-1, Santa Cruz Biotechnology, Heidelberg, Germany), anti-TCRβ (H-197, Santa Cruz Biotechnology, Heidelberg, Germany), anti-CD3ε (M20, Santa Cruz Biotechnology, Heidelberg, Germany) and anti-ζ (a rabbit serum [51]). Immunoblots were developed using HRPO-conjugated secondary antibodies and detected by chemiluminescence on a LAS-4000 mini imager (GE Healthcare, Freiburg, Germany).

### 4.7. Illumination Devices

Two types of illumination devices were used for the different experiments performed in this study, as described earlier [31]. Briefly, the first device was built as a planar light source using an array of red (Osram, LH W5AM, Mouser Electronics, Munich, Germany) and far-red (LZ4-00R308, LED Engin, San Jose, CA, USA) light-emitting diodes (LEDs). This illumination device was used at 50% intensity at a distance of 5 cm, corresponding to approximately 150 µmol/m^2^s, for a duration of 30 s for all PhyBt pre-illumination steps to achieve complete photoconversion.

The second device was built as a cylinder enclosing a flow cytometry tube in the center, surrounded by 37 °C water to keep physiological temperatures and further outside rings of red (Super Bright Red, Kingbright Electronic Europe, Issum, Germany) and far-red (LED740 series, Roithner Lasertechnik, Vienna, Austria) LEDs. The cylindrical illumination device was used for all calcium experiments in combination with a MACSQuant X flow cytometer (Miltenyi Biotec, Bergisch Gladbach, Germany).

The conversion of the 660 nm light intensity values of the cylindrical illumination device to ligand binding half-lives was done as described [31]. Briefly, the PhyB dimer binding half-life τ was determined by the sum of the light-independent off-rate *k_off_* and the light-dependent off-rate *k_i_* by the formula $\tau =\frac{\ln2}{2\left(k_{off}+k_{i}\right)}$. *k_i_* was derived from the *k_off_* using the ratio of the 660 nm light intensity *I* to *I*_0_ with *I*_0_ being the intensity where *k_i_* equals *k_off_* (2%) by the formula $k_{i}=k_{off}\frac{I}{I_{0}}$.

### 4.8. Quantification of TCR Surface Expression

To quantify total amounts of TCR molecules on the cell surface, Quantibrite Beads (Becton Dickinson, Heidelberg, Germany) were utilized. The beads were used as recommended in the manufacturer’s protocol and measured on a Gallios flow cytometer (Beckman Coulter, Krefeld, Germany). Data analysis was done using FlowJo software v10.4.2. Using the amounts of PE molecules per bead and the geometric mean of the measured fluorescence, a linear equation was fitted to the observed data. Jurkat cells were stained with saturating amounts of PE-labelled anti-Vβ3 or anti-Vβ8-PE (both Becton Dickinson, Heidelberg, Germany) antibodies. Geometric means of the antibody stains were entered into the linear equation to calculate amounts of TCR molecules on the cell surface.

For testing Vβ3 and Vβ8 co-expression on the different cell lines, dual staining experiments with anti-Vβ3-biotin (Ancell, Bayport, MN, USA) followed by streptavidin-Pacific blue and anti-Vβ8-PE (Becton Dickinson, Heidelberg, Germany) were performed.

### 4.9. Repetition of Experiments and Data Presentation

In this study: graphs displaying representative experiments, such as for the immunoblots, the calcium traces, or the flow cytometry histograms, “*n*” indicates the number of independent experiments that the depicted results were obtained. Graphs displaying quantified data from multiple experiments, such as the quantified calcium influx, depict individual data points, and average values with their uncertainties shown as the standard error of the mean (SEM). Statistical analysis was performed as described using the software Prism 8 (Graphpad Software, San Diego, CA, USA). Figures were compiled with Illustrator CC (Adobe Inc., San Jose, CA, USA).

## Data Availability

Not applicable.

## Supplementary Materials

The following are available online at https://www.mdpi.com/article/10.3390/ijms22094920/s1, Figure S1: Cross-antagonism when adding the agonist and antagonist simultaneously, Figure S2: The C10 Signal Inhibition model, Figure S3: The KPR Steps Inhibition model, Figure S4: Result of KPR Steps Inhibition model, Table S1: Primers for cloning of pOSY082, Table S2: Categorized list of all parameters of the mathematical models with their default numerical values.
