# Supplementary material for: Cross-TCR Antagonism Revealed by Optogenetically Tuning the Half-Life of the TCR Ligand Binding
Source: Int J Mol Sci. 2021 May 6;22(9):4920. doi: 10.3390/ijms22094920 (PMC8124730; doi:10.3390/ijms22094920)
Supplement: Supplementary file 1 [file ijms-22-04920-s001.zip › ijms-1144395-SI.pdf]

International Journal of
Molecular Sciences

MDPI

Supplemental Information

Cross-TCR Antagonism Revealed by Optogenetically Tuning the Half-Life of the TCR Ligand Binding

Omid Sascha Yousefi, Matias Ruggieri, Vincent Idstein, Kai Uwe von Prillwitz, Laurenz A. Herr, Julia Chalupsky, Maja Köhn, Wilfried Weber, Jens Timmer and Wolfgang W. A. Schamel

1. Supplemental figures S1, S2, S3, S4 and table S1.

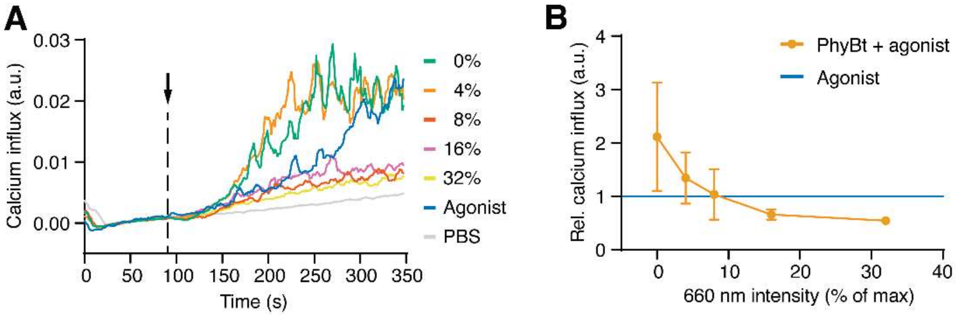

Figure S1. Cross-antagonism when adding the agonist and antagonist simultaneously. (A) JK82 cells were stimulated with 63 nM PhyBt(660) and 50 ng/ml of the agonist anti-Vβ8 at the same time (arrow) while varying the 660 nm light intensity and measuring calcium influx by flow cytometry as in figure 3A. (B) Quantification of three experiments (n=3) was done as in figure 3B. This experiment shows that the PhyBt ligand antagonizes the activatory signal by the anti-Vβ8 antibody and that this occurs only at low binding half-lives (16% and 32% of 660 nm light intensity). In one experiment (not shown) the anti-Vβ8 antibody stimulation resulted in a more rapid and strong calcium influx than seen in (A); in this case antagonism was not observed. Since the agonistic signal was different from the other 3 experiments, we did not include this single experiment in the analysis of (B).

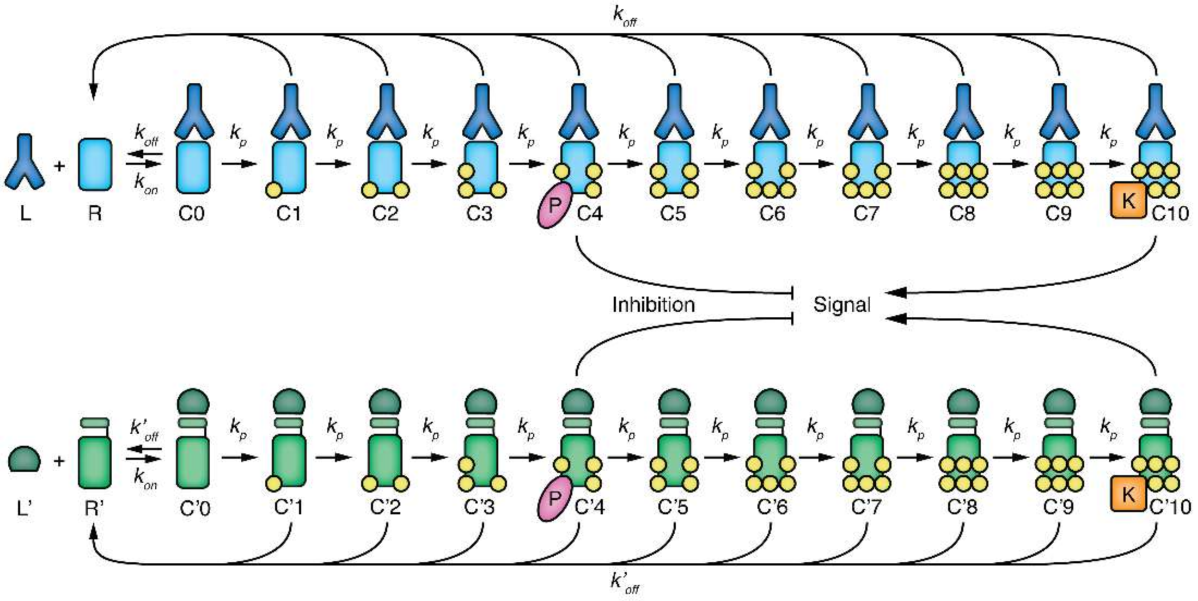

Figure S2. The C10 Signal Inhibition model.Full scheme of Fig. 4A. Two independent KPR models, one for each ligand-receptor pair (top: agonist ligand, bottom: opto-ligand) are combined at the signal level. In each individual KPR model, after ligand (*L* or *L′*) and receptor (*R* or *R′*) bind, the ligand-receptor complex (*C₀* or *C₀′*) has to undergo several modification steps before reaching the final signaling state (*C₁₀* or *C₁₀′*). The modification rate, k_p, which is identical for all KPR steps, and the initial binding rate, k_{on}, are the same for both ligand-receptor pairs. In each state, the ligand-receptor complex can dissociate (with a rate k_{off} or k_{off}' leading to instant reversal of all so far attained modifications. The intermediate state, (C_4 or C_4'), elicits an inhibitory signal that reduces the activatory signal from C_{10} or C_{10}'. Biologically, the inhibitory and activatory signals are related to the activities of a phosphatase *P* and a kinase *K*, respectively. The precise mechanisms are not explicitly modeled.

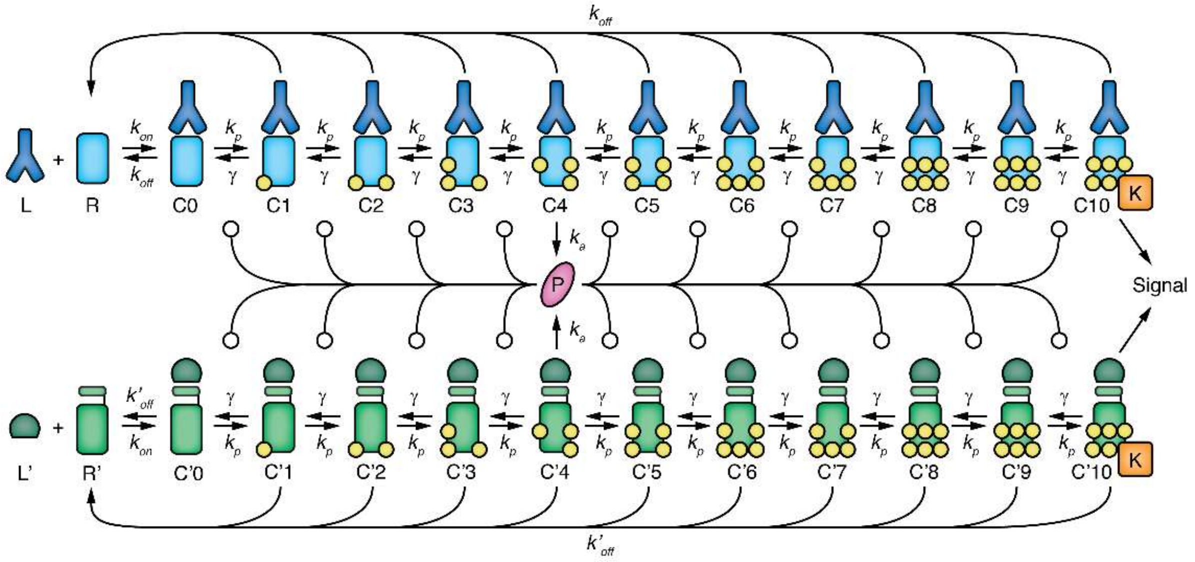

Figure S3. The KPR Steps Inhibition model. This model is a modification of the C10 Signal Inhibition model. The general KPR mechanism and the activatory signal elicited by the state C_{10} or C_{10}' are as before. The inhibition is now modeled via the activation (rate k_a) of a phosphatase (P) which in turn catalyzes the backwards direction of all KPR modifications (intrinsic rate γ). Due to the interaction with the same phosphatase, the kinetics of the two ligand-receptor pairs are now explicitly coupled. The phosphatase is automatically inactivated over time (rate k_d) which is not shown in the figure.

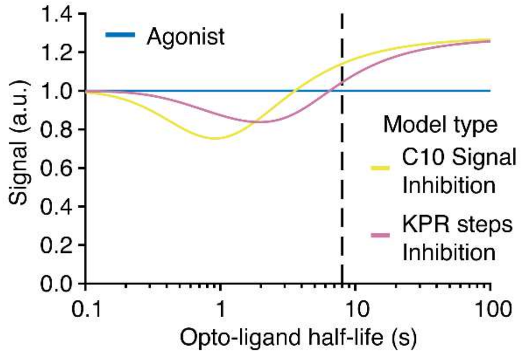

Figure S4. Result of KPR Steps Inhibition model. Comparison of the results (signal vs opto-ligand binding half-life) of the C10 Signal Inhibition model and the KPR Steps Inhibition model (see main text, Fig. 4, S2, S3). The dashed line marks $\tau_{KPR} = 8\ sec$.

Table S1. Primers for cloning of pOSY082.

Primer	Sequence
O197	GGCGGTCACGAACTCCAGAAGGAC
O198	GTCCTTCTGGAGTTCGTGACCGCC
O199	CCTCATACAAATCCATGATAGATTTGGTACGTTGATTATG
O200	CATAATCAACGTACCAAATCTATCATGGATTTGTATGAGG
O204	CAAAAACAAATTACAAAATTCAAAATTTTATCGATACTAGTGGCTCCGGTGCCCGTC

O205	CATGGTGGCTTCGAATTCGCTAGCTCTAGACTGTGTTCTGGCGGCAAACCC
O206	TCTAGAGCTAGCGAATTCGAAGCCACCATGGGATGGAGCTG
O207	TCACAAATTTTGTAATCCAGAGGTTGATTGTCGACGCGGCCGCGGATCCTCAGAAATCCTTTCTCTTG

2. Supplemental description of the mathematical model of antagonism in T cell activation

2.1. *Basic KPR model for single ligand-receptor pair*

The kinetic proofreading model for T cell activation as proposed by McKeithan [1] assumes that T cell receptors do not initiate signaling immediately after ligand binding (association rate k_{on}), but only after a series of N receptor modifications has been completed. For simplicity it is assumed that the rate of the modifications, k_p, as well as the receptor-ligand dissociation rate, k_{off}, are independent of the current receptor state. Importantly, after dissociation, all modifications are essentially instantly reverted, so that after rebinding the series of modifications restarts from the beginning (Fig. 4A and S2). When denoting the concentrations of free ligand by L, unoccupied receptor by R, and bound receptor in the different modification states by C_i, $0 \leq i \leq N$, then the system is governed by the following set of ordinary differential equations,

$$\dot{C}_0 = k_{on}LR - (k_{off} + k_p)C_0, \tag{1a}$$

$$\dot{C}_i = k_pC_{i-1} - (k_{off} + k_p)C_i, \qquad \mathbf{1 \leq \mathit{i} \leq \mathit{N} - 1} \tag{1b}$$

$$\dot{C}_N = k_pC_{N-1} - k_{off}C_N. \tag{1c}$$

An assumption required to describe the system by this set of differential equations is that free ligands disperse quickly, so that free ligands easily find free receptors.

By summing up equations (1a-c) one obtains for the total concentration of bound receptor, $C_T = \sum_{i=0}^{N} C_i$,

$$\dot{C}_T = k_{on}LR - k_{off}C_T. \tag{2}$$

Furthermore, the concentrations of free ligand and receptor are determined via the conversion equations,

$$L = L_T - C_T, \tag{3a}$$

$$R = R_T - C_T, \tag{3b}$$

with fixed total ligand (receptor) concentration L_T (R_T). Equation (2) describes an occupancy model of T cell activation and, after inserting equations (3a, b), leads to a quadratic equation for the equilibrium solution of C_T. Using $K_D = k_{off}/k_{on}$, the biologically meaningful solution of this quadratic equation is,

$$C_T = \frac{1}{2}\left(L_T + R_T + K_D - \sqrt{(L_T + R_T + K_D)^2 - 4L_TR_T}\right), \tag{4}$$

see also [2]. Concerning the concentrations of the different bound receptor states, solving equations (1b, c) for equilibrium yields the relations,

$$C_i = \alpha C_{i-1} = \alpha^i C_0, \qquad \mathbf{0 \le i \le N-1} \qquad (5a)$$

$$C_N = \frac{\alpha}{1-\alpha} C_{N-1} = \frac{\alpha^N}{1-\alpha} C_0, \qquad (5b)$$

where $\alpha = \frac{k_p}{k_{off}+k_p}$ is the probability that a single KPR step is completed before dissociation [1,2]. By using equations (5a, b) together with $C_T = \sum_{i=0}^{N} C_i$ and exploiting the geometric sum $\sum_{i=0}^{N-1} \alpha^i = \frac{1-\alpha^N}{1-\alpha}$, one finds,

$$C_i = (1-\alpha)\alpha^i C_T, \qquad \mathbf{0 \le i \le N-1} \qquad (6a)$$

$$C_N = \alpha^N C_T. \qquad (6b)$$

With $0 < \alpha < 1$ it can be seen from this solution that C_i is a decreasing function of i (for
$0 \le i \le N-1$), meaning that later receptor states are less likely to be reached. Only the final signaling state ($i = N$) can be an exception from this rule since, for large enough α, receptors can accumulate in this state. In the extreme case $\alpha \to 1$ one finds $C_N \to C_T$ and $C_i \to 0$ for all other states.

Due to the KPR mechanism only ligands with sufficient binding time can trigger T cell activation. The corresponding threshold binding time τ_{KPR} (here also referred to as the *KPR time*) can be defined via the condition $C_N = \frac{1}{2} C_T$, meaning that half of the bound receptors are in the active signaling state. Using equation (6b) this condition becomes $\alpha^N = \frac{1}{2}$. With $\alpha = \frac{k_p}{k_{off}+k_p}$ and given k_p one can calculate the corresponding k_{off} and then use $\tau_{KPR} = \frac{\ln(2)}{k_{off}}$ to obtain the KPR time. Conversely, when τ_{KPR} is known one can use the condition $\alpha^N = \frac{1}{2}$ with $k_{off} = \frac{\ln(2)}{\tau_{KPR}}$ to calculate the corresponding KPR step rate k_p. This is in fact the more relevant direction here, since we use $\tau_{KPR} = 8\, s$ from [3] and treat k_p as an otherwise unknown model parameter.

Note that the condition $C_N = \frac{1}{2} C_T$ (or rather $\alpha^N = \frac{1}{2}$) is independent of the total concentrations of ligand and receptor, implying that also τ_{KPR} and its relation to k_p are concentration-independent. Also note, that $C_N = \frac{1}{2} C_T$ does not necessarily mean that the resulting signal is half-maximal because (1) for increasing binding time (decreasing k_{off}) not only the ratio ${C_N}/{C_T}$ but also the total amount of bound receptors, C_T, itself increases, and (2) since additional events in the downstream signaling (e.g., saturation effects) might alter the relation between the final signal and the binding time.

*2.2. Single KPR model with an inhibition signal (C10 Signal Inhibition)***Error! Reference source not found.Error! Reference source not found.Error! Reference source not found.Error! Reference source not found.Error! Reference source not found.Error! Reference source not found.**

As suggested [1] also bound receptors in earlier states might trigger a signal, but potentially one that is qualitatively different from the main signal. Rabinowitz et al. proposed a model with only two modified receptor states where the first state could inhibit the activation signal of the second state [4]. Applying this idea to the basic KPR model introduced above, we here assume that receptors in one or more intermediate states $I \subset$

$\{1,2,\ldots,N-1\}$ trigger an inhibiting signal (Fig. 4A and S2). The arguably simplest way (in a mathematical sense) to implement the inhibition is by subtracting the inhibition signal from the activation signal. Since this inhibition does not involve any feedback to the KPR mechanism itself, we refer to this model as the *C10 Signal Inhibition* model (C10 since in practice we use $N = 10$). When the individual signals are simply assumed to be proportional to the concentrations of the corresponding signaling states, one can write,

$$S_{raw} = C_N - f \cdot \sum_{i \in I} C_i. \tag{7}$$

We denote this signal by S_{raw} since it will later be subject to additional signal processing. The factor f can be used to tune the relative strength between positive and negative signal. Biologically, the positive or negative signal might involve the activity of kinases or phosphatases, such as Lck and ZAP70 or SHP1, respectively [5–10] and since phosphatases typically have a stronger activity than kinases, $f > 1$ should be expected.

In the KPR model without inhibition the threshold binding time τ_{KPR} for triggering a signal was defined via the condition $C_N = \frac{1}{2} C_T$. Including the inhibition, it is in principle possible that we find a signal $S_{raw} < 0$ even though $C_N = \frac{1}{2} C_T$ is satisfied, which contradicts the idea of the KPR time. In order to take the inhibition into account, the defining condition for τ_{KPR} can be generalized to $C_N - f \cdot \sum_{i \in I} C_i = \frac{1}{2} C_T$. With equations (6a, b) this can be rewritten as,

$$\alpha^N - f \cdot \sum_{i \in I} (1-\alpha)\alpha^i = \frac{1}{2}, \text{with } \alpha = \frac{k_p}{\frac{\ln(2)}{\tau_{KPR}} + k_p} \tag{8}$$

For given τ_{KPR} this relation can again be used to calculate the corresponding KPR step rate k_P. It is important to realize that the relation between τ_{KPR} and k_p is still concentration-independent but depends on the number of KPR steps, N, the set of inhibiting states, I, and the inhibition factor, f. If, for example, f is increased (implying a stronger inhibition) then solving equation (8) will result in a larger KPR step rate k_p. Since with larger k_p more receptors reach the final (positive) signaling state, this will to some degree counteract the stronger inhibition caused by the larger factor f.

2.3. C10 Signal Inhibition model with two ligand-receptor pairs

The final T cells in our cross-antagonism experiment exhibit two different types of receptors that bind to two different ligands (Fig. 4A and S2). Adding a second ligand-receptor pair to the C10 Signal Inhibition model results in two independent KPR models, one for each ligand-receptor pair. The important conditions for this independence are (1) the absence of competition between the ligands, since each ligand has its own receptor, and (2) that in the C10 Signal Inhibition model the inhibition is not implemented via a feedback to the KPR steps themselves but instead via an additional contribution to the output of the activating state C_N (C_{10} if $N = 10$). All equations from the previous sections can thus be duplicated for the second ligand-receptor pair. The model output, i.e. the net signal before post-processing, see also equation (7), now reads,

$$S_{raw} = C_N + C'_N - f \cdot \sum_{i \in I} (C_i + C'_i), \tag{9}$$

where the concentrations C'_i, C'_N correspond to the second ligand-receptor pair. For equation (9) it has been assumed that the inhibition factor f and the positions of the inhibiting states, I, are the same for both ligand-receptor pairs.

In the experiments the first ligand is an agonist, i.e., it has sufficient binding time to produce a positive net signal. The binding time of the second ligand can be controlled via illumination with light. We therefore also refer to the two ligands as the *agonist ligand* and the *opto-ligand*. For different binding times, the net signal of the opto-ligand, $C'_N - f \cdot$

$\sum_{i\in I} C_i'$, can be negative, positive, or close to zero. This implies that the opto-ligand can act as an antagonist (effectively reducing the signal induced by the first ligand) or as a co-agonist (enhancing the positive signal) or be non-stimulatory.

2.4. Inhibition model with reversal of KPR steps (KPR Steps Inhibition model)

An alternative to the C10 Signal Inhibition model discussed above, is to allow a reversal of the KPR steps. Following previous publications [2,11], a possible implementation of this idea is that the intermediate, inhibiting receptor states (I) activate a phosphatase (with rate k_a) which in turn induces a backwards reaction rate (γ) of all the KPR steps (Fig. S3). The phosphatase is automatically inactivated over time with rate k_d. Both ligand-and-receptor pairs interact with the same phosphatase which means that the two submodels corresponding to the two ligand-receptor pairs are now explicitly coupled. The underlying biological assumption is that the different receptor types are mixed on the cell surface, either because the receptors are localized within the same nanocluster [12] or because they rapidly diffuse and meet each other. The model output before post-processing is now simply $S_{raw} = C_N + C_N'$, which corresponds to equation (9) with $f = 0$, meaning that we do not use an additional difference inhibition in this model. Since $C_N' \geq 0$, this implies that without the explicit coupling of the submodels the opto-ligand could never act as an antagonist. Denoting the concentration of the activated (total) phosphatase by P (P_T), the model is now governed by the following set of differential equations,

$$\dot{\boldsymbol{C}}_{\boldsymbol{0}}^{(\prime)} = \boldsymbol{k_{on}}\left(\boldsymbol{L_T^{(\prime)}} - \boldsymbol{C_T^{(\prime)}}\right)\left(\boldsymbol{R_T^{(\prime)}} - \boldsymbol{C_T^{(\prime)}}\right) - \left(\boldsymbol{k_{off}^{(\prime)}} + \boldsymbol{k_p}\right)\boldsymbol{C}_{\boldsymbol{0}}^{(\prime)} + \boldsymbol{\gamma P C_1^{(\prime)}}, \tag{10a}$$

$$\dot{C}_i^{(\prime)} = k_p C_{i-1}^{(\prime)} - \left(k_{off}^{(\prime)} + k_p + \gamma P\right)C_i^{(\prime)} + \gamma P C_{i+1}^{(\prime)}, \qquad \boldsymbol{1 \leq i \leq N-1} \tag{10b}$$

$$\dot{C}_N^{(\prime)} = k_p C_{N-1}^{(\prime)} - \left(k_{off}^{(\prime)} + \gamma P\right)C_N^{(\prime)}, \tag{10c}$$

$$\dot{P} = k_a \sum_{i\in I}(C_i + C_i')\,(P_T - P) - k_d P. \tag{10d}$$

The notation, e.g., $\dot{C}_i^{(\prime)}$ means that there is one such quantity or equation for the first and the second ligand-receptor pair each. Apart from that, the notation is as in the previous sections. Parameters associated with the phosphatase (k_a, k_d, γ) as well as k_{on}, k_p, I and N are independent of the considered ligand-receptor pair. Note that when setting the backwards reaction rate $\gamma = 0$ or removing the phosphatase ($P, P_T = 0$) equations (10a-c) reduce to the basic KPR equations (1a-c) (or rather to two duplicates of these equations, one for each ligand-receptor pair). We solve equations (10a-d) by numerical integration over sufficient time so that equilibrium is reached. Likewise, the condition $C_N' = \frac{1}{2}C_T'$ relating the KPR step rate k_p to the threshold binding time τ_{KPR} is solved numerically (for the opto-ligand alone).

2.5. Final signal processing and parameter values

We apply two post-processing steps to the raw model output signal from equation (9) (for the KPR Steps Inhibition model with $f = 0$). First, we truncate negative values, $S_{raw,+} = \max\{S_{raw}, 0\}$, since the final, inhibited signal should never be negative. Second, we apply a saturation function,

$$S = S_{max}\frac{S_{raw,+}}{K_{1/2} + S_{raw,+}}, \tag{11}$$

to make sure that the signal cannot rise indefinitely. Mathematically, the saturation function also implies that even in the C10 Signal Inhibition model the signals of the two

receptor types are not independent anymore. Biologically, the saturation could be explained by the activation of some downstream signaling protein (shared by both receptor types) which is only present in a limited amount. The half-saturation constant in equation (11) we set to $K_{1/2} = 10^{-2}\ nM$ which coincides with the total concentration of the agonist-receptor that is used in the experiments (see also Table 2). Due to the small off-rate of the agonist, almost all agonist-receptors are bound in the final signaling state, implying that the agonist alone produces a half-maximal signal. In order to set the agonist-only signal to $S = 1$ (see Figure 4) we choose $S_{max} = 2$. Since the total concentration of the opto-ligand receptor is smaller than that of the agonist-receptor, full saturation of the joint signal will never be reached. Nevertheless, the saturation function makes sure that the enhancement of the signal in the case where the opto-ligand acts as a co-agonist is reduced.

Concerning the other model parameters, the total concentrations of the ligands and receptors as well as the off-rate k_{off} of the agonist ligand are known from the experimental setup (receptor concentrations have been obtained by multiplying the number of receptors per cell with the concentration of cells per volume and then translating units to nM). Note that these are also the only parameters that differ between the two ligand-receptor pairs. The threshold binding time $\tau_{KPR} = 8s$ is taken from our earlier optogenetic publication [3]. In principle, it is also possible to extract the on-rate k_{on} from this paper but we did not do so, since the models used here neglect the bivalent nature of the ligand-receptor binding used earlier [3].

Table 2. Categorized list of all parameters of the mathematical models with their default numerical values. Parameters that do not have a primed version are the same for both ligand-receptor pairs. For $\boldsymbol{k_p}$ the value in the brackets is the one used by the KPR Steps Inhibition model for the corresponding line in Figure 4G.

Type	Parameter [unit]	Default value	Remark
Concentrations	$L_T\ [nM]$	333	From experimental setup
	$L'_T\ [nM]$	63	From experimental setup
	$R_T\ [nM]$	10^{-2}	From experimental setup
	$R'_T\ [nM]$	$7.58 \cdot 10^{-3}$	From experimental setup
General kinetic parameters	$k_{off}\ [s^{-1}]$	10^{-3}	From experimental setup
	$k'_{off}\ [s^{-1}]$	$10^{-1} \leq \frac{\ln(2)}{k'_{off}} \leq 10^{2}$	x-axis in Fig. 4B-G, S4
	$k_{on}\ [s^{-1}\ nM^{-1}]$	$8 \cdot 10^{-3}$	-
	$k_p\ [s^{-1}]$	4.74 (2.37)	Calculated
Other KPR parameters	$\tau_{KPR}\ [s]$	8	From Yousefi et al., 2019
	N	10	-
	i	4	-
C10 Signal Inhibition model	f	20	-
KPR Steps Inhibition model	$k_a\ [s^{-1}\ nM^{-1}]$	500	-
	$k_d\ [s^{-1}]$	1	-
	P_T	1	-
	$\gamma\ [s^{-1}]$	5	-
Saturation	S_{max}	2	-
	$K_{1/2}\ [nM]$	10^{-2}	-

If possible, unknown parameters have been chosen in a biologically meaningful way. For example, the parameters specific to the KPR Steps Inhibition model (see Table 2) haven been chosen such that, when the half-life of the opto-ligand is in the antagonistic regime, forward and backward reaction rate of the KPR steps are of similar magnitude. Parameters that are not known from the experimental setup and that cannot be deduced otherwise, are chosen in a way that the model results resemble the experimental results, i.e., that there is a moderate inhibition effect induced by the opto-ligand at intermediate

binding times. Additionally, variation of some unknown parameters (mainly the position and number of the inhibitory states) showed a qualitative robustness of the model results (D, E, G). The default parameters used in Figure 4 (B, F) are presented in Table 2.

2.6. Modelling software

All numerical calculations and simulations of the model, as well as plotting, have been performed in MATLAB R2019b.

References

1. McKeithan, T.W. (1995). Kinetic proofreading in T-cell receptor signal transduction. Proc Natl Acad Sci U S A *92*, 5042-5046.
2. Lever, M., Maini, P.K., van der Merwe, P.A., and Dushek, O. (2014). Phenotypic models of T cell activation. Nat Rev Immunol *14*, 619-629.
3. Yousefi, O.S., Gunther, M., Horner, M., Chalupsky, J., Wess, M., Brandl, S.M., Smith, R.W., Fleck, C., Kunkel, T., Zurbriggen, M.D., *et al.* (2019). Optogenetic control shows that kinetic proofreading regulates the activity of the T cell receptor. eLife *8*, e42475.
4. Rabinowitz, J.D., Beeson, C., Lyons, D.S., Davis, M.M., and McConnell, H.M. (1996). Kinetic discrimination in T-cell activation. Proc Natl Acad Sci U S A *93*, 1401-1405.
5. Chakraborty, A.K., and Weiss, A. (2014). Insights into the initiation of TCR signaling. Nat Immunol *15*, 798-807.
6. Chan, A.C., Iwashima, M., Turck, C.W., and Weiss, A. (1992). ZAP-70: a 70 kd protein-tyrosine kinase that associates with the TCR zeta chain. Cell *71*, 649-662.
7. Courtney, A.H., Lo, W.L., and Weiss, A. (2018). TCR Signaling: Mechanisms of Initiation and Propagation. Trends Biochem Sci *43*, 108-123.
8. Dittel, B.N., Stefanova, I., Germain, R.N., and Janeway, C.A. (1999). Cross-antagonism of a T cell clone expressing two distinct T cell receptors. Immunity *11*, 289 - 298.
9. Kilgore, N.E., Carter, J.D., Lorenz, U., and Evavold, B.D. (2003). Cutting Edge: Dependence of TCR Antagonism on Src Homology 2 Domain-Containing Protein Tyrosine Phosphatase Activity. J Immunol *170*, 4891 - 4895.
10. Stefanova, I., Hemmer, B., Vergelli, M., Martin, R., Biddison, W.E., and Germain, R.N. (2003). TCR ligand discrimination is enforced by competing ERK positive and SHP-1 negative feedback pathways. Nat Immunol *4*, 248 - 254.
11. Francois, P., Voisinne, G., Siggia, E.D., Altan-Bonnet, G., and Vergassola, M. (2013). Phenotypic model for early T-cell activation displaying sensitivity, specificity, and antagonism. Proc Natl Acad Sci U S A *110*, E888-897.
12. Schamel, W.W., Arechaga, I., Risueno, R.M., van Santen, H.M., Cabezas, P., Risco, C., Valpuesta, J.M., and Alarcon, B. (2005). Coexistence of multivalent and monovalent TCRs explains high sensitivity and wide range of response. J. Exp. Med. *202*, 493-503.
